# Seven-Year Experience of Intramural Surgery in the Middle East: A Safety and Feasibility Analysis

**DOI:** 10.3390/jcm13133989

**Published:** 2024-07-08

**Authors:** Gabriela Restrepo-Rodas, Juan S. Barajas-Gamboa, Jerry T. Dang, Maja I. Piechowska-Jóźwiak, Mohammed Khan, Gabriel Diaz Del Gobbo, Mohammed Abdallah, Cristobal Moreno, Carlos Abril, Juan Pablo Pantoja, Alfredo D. Guerron, Ricard Corcelles, Matthew Kroh, John Rodriguez

**Affiliations:** 1 Department of General Surgery, Cleveland Clinic Abu Dhabi, Abu Dhabi 112412, United Arab Emirates; restreg@clevelandclinicabudhabi.ae (G.R.-R.); barajaj@clevelandclinicabudhabi.ae (J.S.B.-G.); piechowskamaja@gmail.com (M.I.P.-J.); 100049726@ku.ac.ae (M.K.); gabodelgobbo@hotmail.com (G.D.D.G.); abdallm2@clevelandclinicabudhabi.ae (M.A.); digestivoalbandea@gmail.com (C.M.); abrilc@clevelandclinicabudhabi.ae (C.A.); pantojj@clevelandclinicabudhabi.ae (J.P.P.); guerrod@clevelandclinicabudhabi.ae (A.D.G.); rodrigj2@clevelandclinicabudhabi.ae (J.R.); 2 Department of General Surgery, Cleveland Clinic, Cleveland, OH 44195, USA; corcelr@ccf.org (R.C.); krohm@ccf.org (M.K.); 3 School of Medicine, Case Western Reserve University, Cleveland, OH 44106, USA

**Keywords:** intramural surgery, E-POEM, G-POEM, Z-POEM, achalasia, gastroparesis, Zenker’s diverticulum

## Abstract

**Background:** Intramural surgery techniques, particularly esophageal peroral endoscopic myotomy (E-POEM), gastric peroral endoscopic myotomy (G-POEM), and peroral endoscopic myotomy for Zenker’s (Z-POEM), have emerged as forefront minimally invasive endoscopic procedures. While several studies have reported on the outcomes in North America and Asia, evidence in the Middle East and North Africa remains limited. This study aims to evaluate the feasibility and safety of intramural surgery techniques within this region. **Methods:** This retrospective cohort study was conducted with approval from the institutional review board. All patients who underwent esophageal peroral endoscopic myotomy, gastric peroral endoscopic myotomy, and peroral endoscopic myotomy for Zenker’s from January 2016 to August 2023 were included. **Results:** In total, 119 patients underwent intramural surgery procedures during this period. The esophageal peroral endoscopic myotomy group had 81 (68%) patients, the gastric peroral endoscopic myotomy had 34 (28.6%) patients, and the peroral endoscopic myotomy for Zenker’s had 4 (3.4%) patients. The full cohort was 48.7% female, with a mean overall age of 40.5 years. The mean overall body mass index was 27.5 kg/m^2^. The chief complaint was dysphagia (*n* = 80, 67.2%). All cases were successfully completed endoscopically. During the first 30 days, the most common complications were nausea/vomiting requiring admission (*n* = 4, 4.76%) and pneumomediastinum (*n* = 2, 2.38%). At a follow-up of 19 months, there were four mortalities; the causes of death were cardiac arrest (three cases) and end-stage prostate cancer (one case). **Conclusions:** Intramural surgery techniques are safe and technically feasible with low complication rates. Our study suggests that clinical success in the Middle East and Northern Africa population is comparable to larger international series.

## 1. Introduction

Over the past decade, there have been significant advancements in endoscopic techniques for treating gastrointestinal motility disorders (GMDs) and Zenker’s diverticula. These disorders have gained increasing attention due to their impact on quality of life. The estimated prevalence varies, with numbers ranging from 1.8 to 12.6 per 100,000 people [1,2,3] for achalasia, 13.8 to 21.5 per 100,000 people [4,5] for gastroparesis, and 2 per 100,000 people [6] for Zenker’s diverticula. Treatment options remain diverse, from pharmaceutical to surgical procedures; consequently, new endoscopic techniques have also been explored. Intramural surgery techniques, such as esophageal peroral endoscopic myotomy (E-POEM), gastric peroral endoscopic myotomy (G-POEM), and peroral endoscopic myotomy for Zenker’s diverticula (Z-POEM), have emerged as forefront minimally invasive procedures.

For achalasia, the injection of botulinum toxin has been approved as a temporary solution due to its frequent relapse and the need for further interventions [7]. Standard procedures like pneumatic dilation or laparoscopic heller myotomy (LHM) are techniques that offer symptomatic relief in 80% and 85% of patients, respectively [8,9]. Originally performed in 2008 by Professor Inoue, E-POEM offers various advantages [10]. It allows for the arbitrary control of the direction and length of the incision on the muscle layer [11]. When compared with LHM, E-POEM showed a clinical response rate of 98% for patients with achalasia type III [12].

For gastroparesis, prokinetics like metoclopramide have been used since the 1970s [13], but concerns have arisen regarding its association with movement disorders and cardiac events [14,15,16]. Therefore, while gastroparesis has a better response rate to conservative therapies, those with a refractory disease usually need pylorus-directed therapies such as the Heineke–Mikulicz pyloroplasty [17]. Another option is G-POEM, also known as peroral pyloromyotomy (POP), first reported by Dr. Khashab in 2013 [18]. G-POEM has been proven to be a safe alternative with few adverse events and short hospital stays for patients with gastroparesis [19,20]. 

For Zenker’s diverticula, the management depends on the size of the defect. Myotomy alone may be recommended for diverticula less than 1 cm, while larger defects may require diverticulectomy or diverticulopexy with myotomy [21]. Peroral endoscopic myotomy for Zenker’s (Z-POEM) was first performed by Ishioka et al. in 1995 [22,23]. Z-POEM has proven to be more effective than rigid endoscopy as it does not need neck hyperextension and has a clinical success rate of nearly 100% in patients with dysphagia [23]. 

While numerous studies have reported outcomes for E-POEM, G-POEM, and Z-POEM in North America and East Asia [24,25,26], there is limited information concerning applicability and outcomes in the Middle East and North Africa (MENA) population. Therefore, the primary aim of this study was to perform a comprehensive evaluation of the feasibility and safety of E-POEM, G-POEM, and Z-POEM for the management of GMDs and Zenker’s diverticula within the MENA region. 

## 2. Materials and Methods

Study design and ethical approvals:

This retrospective study was conducted between January 2016 and August 2023. We reviewed electronic medical records to collect data on demographic characteristics, preoperative comorbidities, and intraoperative and postoperative outcomes. All patients who underwent either E-POEM, G-POEM, or Z-POEM during this time frame were included. This study was approved by our institution’s Research Ethics Committee (REC) under the internal number A-2019-050.

Objectives:

This study aimed to evaluate the feasibility and safety of E-POEM, G-POEM, and Z-POEM for managing GMDs and Zenker’s diverticula at a tertiary referral academic medical center in the United Arab Emirates. 

Population: Patients with a diagnosis of GMDs, including achalasia, gastroparesis, and Zenker’s diverticula. Patients were divided into three groups based on the procedure: those who underwent E-POEM, G-POEM, and Z-POEM. 

Indication for the procedures: To be scheduled for E-POEM, patients had to have a confirmed diagnosis of achalasia by high-resolution esophageal manometry. To be scheduled for G-POEM, patients had to have a confirmed diagnosis of gastroparesis by 4 h gastric emptying scintigraphy. To be scheduled for Z-POEM, patients had to have evidence of Zenker’s diverticulum in fluoroscopy and upper endoscopy. 

Inclusion and exclusion criteria:

Patients diagnosed with achalasia, gastroparesis, or Zenker’s diverticulum that underwent E-POEM, G-POEM, and Z-POEM between January 2016 and August 2023 were included. Patients deemed to have a mechanical obstruction were excluded from the analysis.

Preoperative management:

Preoperative assessment differed according to the procedure (Table 1). Patients in this study received a thorough evaluation to reach a diagnosis and define the treatment course. All patients underwent an upper endoscopy to delineate the anatomy, exclude malignancy, and evaluate the mucosal tissue.

For E-POEM, symptomatic evaluation began by considering the severity of the symptoms. Then, esophageal manometry was performed to classify achalasia into different categories: elevated lower esophageal sphincter pressure (LES), incomplete LES relaxation, and absence of normal peristaltic contractions [27]. A barium esophagogram was ordered in certain patients who needed further evaluation of the distal esophageal morphology. 

For G-POEM, the evaluation began by assessing the referred symptoms, followed by gastric emptying by scintigraphy utilized to define the severity of the disease. 

For Z-POEM, an esophagogram was performed to highlight the features of the diverticulum. Esophageal scintigraphy was also performed in patients who needed further assessment of the motility and stagnation at the level of the diverticulum [28]. 

In all cases, the need for additional imaging tests was assessed individually. All patients maintained a liquid diet for 48 h before the procedure. 

**Figure 1:**
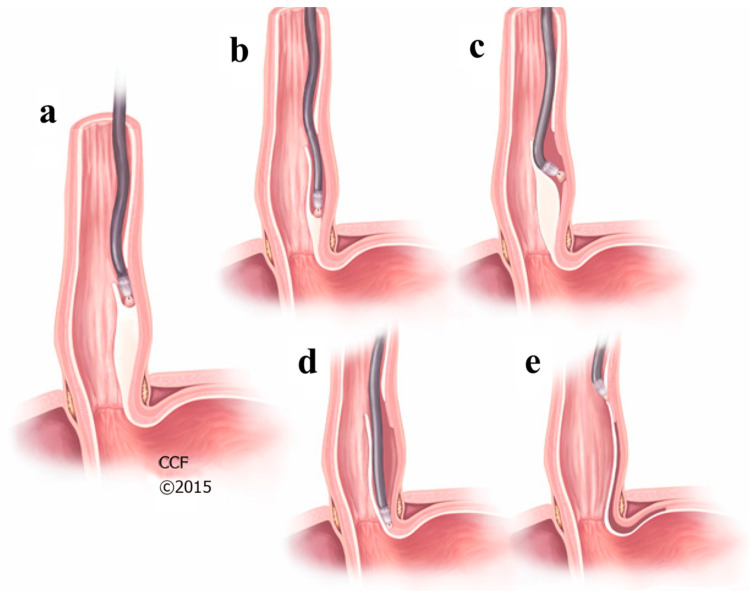
E-POEM technique. (**a**) Submucosal injection in the mid esophagus to create a submucosal bleb. (**b**) Creation of submucosal tunnel. (**c**,**d**) Esophagogastric myotomy. (**e**) Closure of entry point with endoscopic clips. (Cleveland Clinic Center for Medical Art & Photography © 2015–2020. All rights reserved).

E-POEM Technique:

All procedures were performed in the operating room under general anesthesia with endotracheal intubation. Patients were placed in decubitus supine position. A diagnostic endoscopy was performed on all patients to assess baseline anatomy and plan accordingly. To begin the E-POEM, the site for the initial submucosal injection and mucosal entry point was chosen following the esophageal lumen orientation, located 10 to 15 cm proximal to the EG junction (Figure 1) [29]. Then, a submucosal bleb was raised at the selected place with a methylene blue dye solution and sodium chloride. After the bleb formation, a mucosal incision was performed, avoiding a full-thickness cut to reduce the risk of mediastinal or peritoneal leakage. The incision allowed for the creation of the submucosal tunnel which could be navigated with a combination of dissection and CO_2_ insufflation [11]. Different techniques, such as finding landmarks or using a radio-opaque clip and fluoroscopy, were used to ensure proper extension of the tunnel and myotomy. Then, the circular muscular layer of the muscularis propia was divided, preserving the longitudinal muscle fibers. Finally, the mucosal flap was closed using hemostatic clips, assuring alignment of the mucosal edges. Patients were observed for complications overnight, and an upper gastrointestinal contrast study was performed on postoperative day 1 in most cases to ensure there were no leaks or obstruction. 

**Figure 2:**
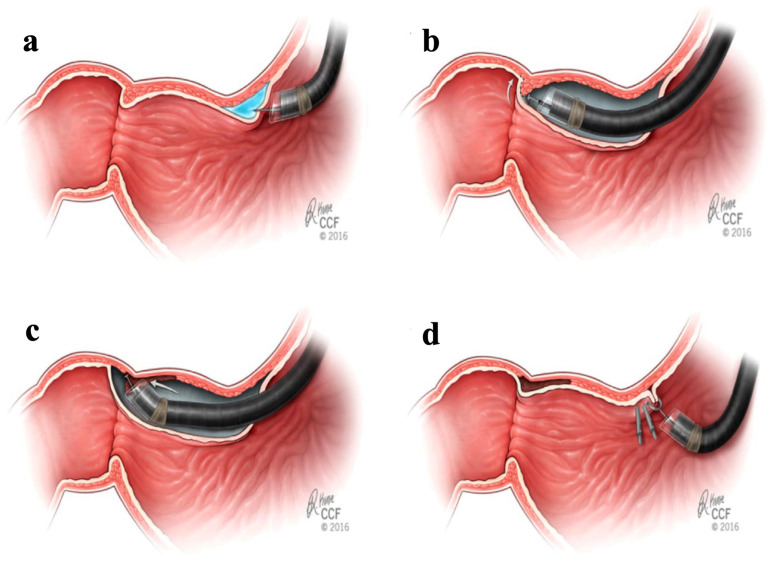
G-POEM technique. (**a**) Creation of the submucosal bleb proximal to the pylorus and mucosotomy. (**b**) Dissection of the submucosa until the pylorus. (**c**) Distal to proximal pyloromyotomy. (**d**) Closure of the mucosotomy by endoscopic clips. (Cleveland Clinic Center for Medical Art & Photography © 2015–2020. All rights reserved).

G-POEM Technique:

All procedures were performed in the operating room under general anesthesia with endotracheal intubation. A diagnostic endoscopy was performed on all patients to assess baseline anatomy and plan accordingly. The gastric peroral endoscopic myotomy followed the same general steps as the E-POEM procedure, with a few key differences. The site for the initial submucosal injection and mucosal entry point was chosen approximately 5 cm from the pylorus along the lesser curvature of the stomach, after which the submucosal bleb was raised using the same solution (Figure 2) [30]. Once the initial incision was performed, the submucosal tunnel was created with careful dissection and CO_2_ insufflation [29]. The myotomy divided the pylorus completely onto the duodenum, as well as 1 cm of gastric antrum adjacent to the pylorus. Finally, the flap was closed using hemostatic clips, and patients were admitted to the hospital for observation. 

**Figure 3:**
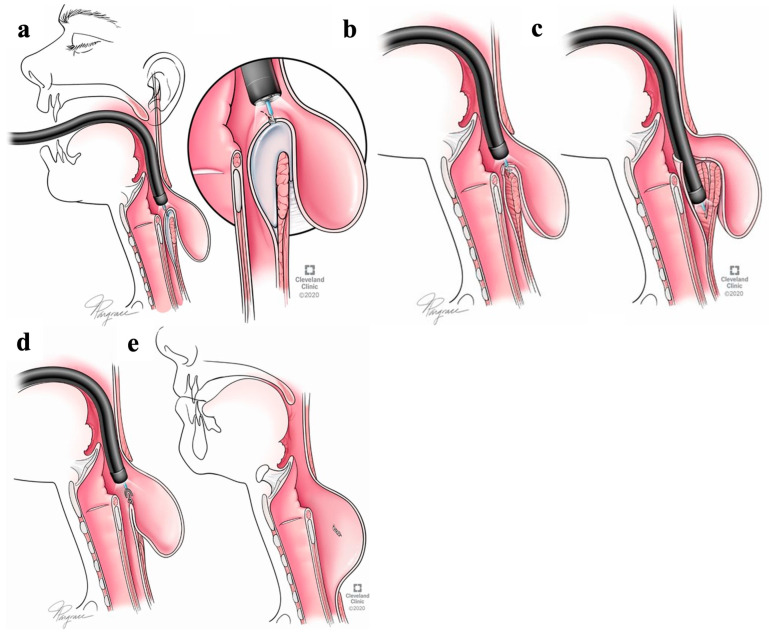
Z-POEM technique. (**a**) Mucosal incision proximal to the septum. (**b**) Creation of submucosal tunnel. (**c**) Dissection of cricopharyngeal muscle fibers. (**d**,**e**) Closure of the entry site with endoscopic clips. (Cleveland Clinic Center for Medical Art & Photography © 2015–2020. All rights reserved).

Z-POEM technique:

All procedures were performed in the operating room under general anesthesia with endotracheal intubation. The endoscope was positioned under direct vision to expose the diverticular septum. The site for the initial incision was 3 cm proximal to the septum or overlying the septum (Figure 3) [29]. Then, a 3 cm submucosal tunnel was performed, followed by division of the septum between the esophagus and the diverticulum where the cricopharyngeal muscle lies [11]. After completing the myotomy, the mucosa was approximated with endoscopic clips. Some patients underwent fluoroscopic examination on postoperative day 1 prior to oral intake. 

Data Collection and Statistical Analysis:

Data collection of the institutional medical records was retrospectively reviewed. The data included but were not limited to patient demographics, indications for the procedure, previous interventions, intraoperative findings, operative times, length of hospitalization, reoperation, operative complications, and mortality. Descriptive statistics were computed for all variables. Frequencies and percentages were calculated for categorical variables, while mean, median, and standard deviation were calculated for quantitative variables. Comparisons were completed using parametric or non-parametric methods where appropriate, and a significance level of *p* < 0.05 was used to determine statistical significance. The comparison test included “independent sample *t* tests” when examining continuous variables, and “Fisher’s exact test” when examining dichotomous variables. All analyses were carried out using R (version 2.13 or higher, The R Foundation for Statistical Computing, Vienna, Austria).

## 3. Results

A total of 119 patients underwent IMS procedures and were included in the study. Out of these, 81 (68%) patients underwent E-POEM, 34 (28.6%) patients underwent G-POEM, and 4 patients (3.4%) underwent Z-POEM. The cohort was 48.7% female, with a mean overall age of 40.5 years (Table 2). The mean overall body mass index (BMI) was 27.5 kg/m^2^. The most common comorbidities in this cohort included GERD (*n* = 36, 30.3%), hypertension (*n* = 23, 19.3%), hyperlipidemia (*n* = 17, 14.3%), and diabetes mellitus (*n* = 16, 13%).

The most common prevalent chief complaint among the cohort was dysphagia (*n* = 80, 67.2%), followed by unintentional weight loss (*n* = 61, 51.2%), heartburn/chest pain (*n* = 56, 47.1%), and regurgitation (*n* = 51, 42.9%).

Among E-POEM patients, types of achalasia were type 1 (*n* = 12, 14.8%), type 2 (*n* = 50, 61.7%), type 3 (*n* = 5, 6.2%), and unspecified (*n* = 14, 17.3%). The etiology of gastroparesis was idiopathic (*n* = 26, 76.5%), diabetes (*n* = 5, 14.7%), and postsurgical (*n* = 3, 8.8%). Previous interventions in the E-POEM group included endoscopic balloon dilation (*n* = 22, 30.9%), botulinum toxin injections (*n* = 10, 14.0%), and Heller–Dor operations (*n* = 7, 9.8%). Previous interventions in the G-POEM group included intrapyloric botulinum toxin injections (*n* = 1, 3.8%). In the last patient group, one patient underwent a cricopharyngeal stapled myotomy prior to Z-POEM (Table 3).

All cases were successfully completed endoscopically. The median operative times were 75.6 ± 27 min for E-POEM, 36.7 ± 22 min for G-POEM, and 28 ± 14 min for Z-POEM (Table 4). Complications within 30 days in the E-POEM group included nausea/vomiting requiring readmission (*n* = 2, 2.5%), pneumomediastinum (*n* = 2, 2.5%), leak (*n* = 1, 1.2%), and gastric perforation (*n* = 1, 1.2%). The G-POEM group included nausea/vomiting requiring readmission (*n* = 2, 5.9%). Patients with Zenker’s diverticulum did not experience any complications. The median overall hospital stay was 2.3 ± 3.2 days. Four mortalities were observed after an extended postprocedural period averaging 19 months. Importantly, these fatalities were not associated with the E-POEM, G-POEM, or Z-POEM interventions. The documented causes of death were as follows: cardiac arrest (three cases) and end-stage metastatic prostate cancer (one case) (Table 5).

## 4. Discussion

Intramural surgery (IMS) represents a notable advancement in minimally invasive procedures in gastrointestinal surgery. In this context, this study constitutes one of the largest investigations in the region, focusing on the mid-term outcomes of E-POEM, G-POEM, and Z-POEM. Several challenges had to be addressed to determine the feasibility of establishing an IMS program. First, access to specialized endoscopic instrumentation and technology. Second, recruitment of skilled surgeons with training and experience in endoscopic surgery. Third, adequate diagnostic and preoperative assessment of these relatively rare disorders [11].

The efficacy of the study was defined by determining the clinical success rate of each procedure. Our study’s success rates are comparable with outcomes reported in different parts of the world. Tan et al.’s systematic review on the safety of E-POEM reported a pooled clinical success of 90.8%, which parallels our E-POEM group’s initial symptom improvement of 90% [31]. Mekaroonkamol et al.’s review of G-POEM outcomes showed a clinical success range of 73 to 100%, consistent with our initial symptom improvement of 88% [32]. Although Z-POEM data are limited, De-la-Morena et al. reported a clinical response rate above 90%, similar to the 100% observed in our Z-POEM cohort despite the limited sample size [33].

All cases were completed endoscopically, yielding a technical success rate of 100%, which aligns with the success rates reported by other authors [31,32,33]. The advantage of reduced operative time was evident. Our study showed a mean operative time of 75.6 ± 27 min for E-POEM, comparable to that reported by Costantini et al. in their E-POEM group [34]. Notably, both are below the average 95 min reported by the same authors for the laparoscopic Heller–Dor group. For G-POEM, we found a mean operative time (36.7 ± 22 min) similar to that reported by Rodriguez et al. (33.8 ± 21 min), [30] whereas the four cases in our Z-POEM group revealed a slightly shorter operative time (28 min) than the time previously reported in a similar cohort (36 min) [35]. Another important factor involves the length of the myotomy, as it has been associated with postoperative complications. Benias et al. conducted a retrospective analysis of 103 patients who underwent E-POEM and concluded that a longer length of the esophageal myotomy (7.2 vs. 8.5 cm, *p* < 0.0068) is a significant factor associated with postprocedural pain requiring admission [36]. Even though our patients in the E-POEM group received an esophageal myotomy longer than the estimated in the mentioned study (9.9 ± 1.7 cm), postoperative pain was not a complication documented in our cohort.

Safety evaluation in our study revealed a low complication rate across all groups, presenting a favorable comparison to the existing literature. Of 81 patients in our E-POEM group, 6 exhibited complications (*n* = 6, 7.5%). We report a lower complication rate than Tefas et al. (13.23%), although this difference can be due to our relatively smaller sample size [37]. To evaluate if demographic differences could have affected the complication rate within the E-POEM group, we compared BMI, age, and comorbidities in patients with and without complications. We observed no statistically significant difference between both groups (*p* > 0.05). However, due to our study’s sample size, further investigation with a larger cohort is necessary to ascertain whether these variables can significantly influence the outcomes of this procedure. Similar to the findings reported by Rodriguez et al., the G-POEM group did not experience intraoperative bleeding; in fact, the only complaint after the procedure was nausea and vomiting (*n* = 2, 5.9%) [30]. Lastly, the Z-POEM group did not present any complications, which is comparable to the complication rate reported by Fan et al., which was also 0% [35]. Although the safety and efficacy of Z-POEM appear promising, it is important to acknowledge that the small sample size for this group may limit the generalizability of these results, necessitating cautious interpretation. The low complication rate (0–5.9%) reported in this study is encouraging as it proves that these procedures can be safely performed within our region. This affirmation is rooted in our center’s resources, featuring surgeons with fellowship training in surgical endoscopy, adherence to international standards of practice, and equipment with the necessary technology for an IMS program.

Several limitations must be considered for interpreting our outcomes and extrapolating the results. First, this was a single-center retrospective study, which introduces potential issues like selection bias, confounding variables, and limited medical record data. Second, it had a small sample size with limited statistical power. Third, the evaluation of symptom resolution lacked standardization across procedures and relied on subjective patient reports. Fourth, the study lacked a follow-up comparison to a preoperative evaluation conducted through clinical imaging or quality-of-life assessments. Larger studies are needed to validate these data and optimize the creation of new IMS programs within the region. Additionally, studies with a longer follow-up period are necessary to evaluate long-term outcomes. The establishment of IMS training programs in this part of the world will expand treatment options available for the local population.

The results presented in this study align with those published in different regions. This enables us to conclude that initiating an IMS program in the region is both safe and feasible.

## 5. Conclusions

In conclusion, intramural surgery and advanced endoscopic techniques are safe and feasible within our region. The clinical success observed in our population aligns with the findings from international studies conducted in various other populations. This encouraging initial experience emphasizes the need for investment in technology and training in intramural surgery. Establishing IMS programs within the region will allow the optimal application of these innovative approaches in the Middle East.

## Figures and Tables

**Table 1:** Preoperative assessment.

Preoperative Assessment			
Procedure	E-POEM	G-POEM	Z-POEM
Imaging	Esophageal manometry	Upper Endoscopy	Barium esophagogram
	Upper endoscopy	Gastric emptying by scintigraphy	Upper Endoscopy
	Contrast fluoroscopy		Esophagogeal scintigraphy with 99 mTc

Abbreviations: (E-POEM) esophageal peroral endoscopic myotomy, (G-POEM) gastric peroral endoscopic myotomy, (Z-POEM) peroral endoscopic myotomy for Zenker’s.

**Table 2:** Demographics and baseline characteristics.

Study Demographics	N:119
Age (Mean, SD, Years)	40.51 ± 16.1
Sex (Female, %)	48.7%
Sex (Male, %)	51.3%
BMI (Mean, SD, kg/m^2^)	27.52 ± 6.4
Comorbidities	
GERD (*n*, %)	(36) 30.3%
Hypertension (*n*, %)	(23) 19.3%
DM2 (*n*, %)	(16) 13%
Hyperlipidemia (*n*, %)	(17) 14.3%
Symptoms	
Constipation (*n*, %)	(11) 9.2%
Dysphagia (*n*, %)	(80) 67.2%
Weight loss (*n*, %)	(61) 51.2%
Regurgitation (*n*, %)	(51) 42.9%
Reflux (*n*, %)	(34) 28.6%
Respiratory symptoms (*n*, %)	(7) 5.9%
Vomiting (*n*, %)	(35) 29.4%
Heartburn, chest pain (*n*, %)	(56) 47.1%

Abbreviations: (BMI) body mass index, (GERD) gastroesophageal reflux disease, (DM2) diabetes mellitus type 2.

**Table 3:** Preoperative assessment: etiology and previous management.

Procedure	E-POEM		G-POEM		Z-POEM	
Number of Cases	81		34		4	
Type/Etiology	Achalasia		Gastroparesis		Zenker’s Diverticulum	
	Type I (*n*, %)	(12) 14.8%	Idiopathic (*n*, %)	(26) 76.5%	-	-
	Type II (*n*, %)	(50) 61.7%	DM2 (*n*, %)	(5) 14.7%		
	Type III (*n*, %)	(5) 6.2%	Postsurgical (*n*, %)	(3) 8.8%		
	Unspecified (*n*, %)	(14) 17.3%	-	-		
Previous Interventions	Endoscopic balloon dilation (*n*, %)	(22) 30.9%	Intrapyloric Botulinum toxin (*n*, %)	(1) 3.8%	Cricopharyngeal myotomy (*n*, %)	(1) 25%
	Botulinum toxin (*n*, %)	(10) 14%	-	-	-	-
	Heller–Dor operation (*n*, %)	(8) 8.8%				

Abbreviations: (E-POEM) esophageal peroral endoscopic myotomy, (G-POEM) gastric peroral endoscopic myotomy, (Z-POEM) peroral endoscopic myotomy for Zenker’s, (DM2) diabetes mellitus type 2.

**Table 4:** Intraoperative findings.

E-POEM		G-POEM		Z-POEM	
Submucosal Tunnel Length in cm (Mean, SD)	17.1 ± 1.9	Mucosectomy Site (Site, %)	Lesser curve (100%)	Septum Length in cm(Mean, SD)	4 ± 0
Myotomy Length–Esophageal in cm (Mean, SD)	9.9 ± 1.7	Myotomy Length in cm (Mean, SD)	3.12 ± 0.65	-	-
Myotomy Length–Stomach in cm (Mean, SD)	3.2 ± 0.45	-	-	-	-
Myotomy Length in cm (Total)	13.2 ± 1.75	-	-	-	-

Abbreviations: (E-POEM) esophageal peroral endoscopic myotomy, (G-POEM) gastric peroral endoscopic myotomy, (Z-POEM) peroral endoscopic myotomy for Zenker’s, (cm) centimeters, (SD) standard deviation.

**Table 5:** Postoperative outcomes.

Procedure	E-POEM	G-POEM	Z-POEM
Mean Operative Time	75.6 ± 27 min	36.7 ± 22 min	28 ± 14 min
Complications			
Nausea/Vomiting (*n*, %)	(2) 2.5%	(2) 5.9%	-
Pneumomediastinum (*n*, %)	(2) 2.5%	-	-
Leaks (*n*, %)	(1) 1.2%	-	-
Gastric Perforation (*n*, %)	(1) 1.2%	-	-
Initial symptom improvement	(73) 90%	(30) 88%	(4) 100%
Length of hospital stay	2.3 ± 3.2 days		
Follow-up months	19.2 ± 22.1 months		

Abbreviations: (E-POEM) esophageal peroral endoscopic myotomy, (G-POEM) gastric peroral endoscopic myotomy, (Z-POEM) peroral endoscopic myotomy for Zenker’s.

## Data Availability

The data that support the findings of this study are available on request from the corresponding author.
